# A reliable and flexible gene manipulation strategy in posthatch zebra finch brain

**DOI:** 10.1038/srep43244

**Published:** 2017-02-24

**Authors:** Somayeh Ahmadiantehrani, Sarah E. London

**Affiliations:** 1 Department of Psychology, Institute for Mind and Biology, Grossman Institute for Neuroscience, Quantitative Biology and Human Behavior, University of Chicago, Chicago, IL USA

## Abstract

Songbird models meaningfully contribute to many fields including learned vocal communication, the neurobiology of social interactions, brain development, and ecology. The value of investigating gene-brain-behavior relationships in songbirds is therefore high. Viral infections typically used in other lab animals to deliver gene editing constructs have been less effective in songbirds, likely due to immune system properties. We therefore leveraged the *in vivo* electroporation strategy used *in utero* in rodents and *in ovo* in poultry, and apply it to posthatch zebra finch songbird chicks. We present a series of experiments with a combination of promoters, fluorescent protein genes, and piggyBac transposase vectors to demonstrate that this can be a reliable, efficient, and flexible strategy for genome manipulation. We discuss options for gene delivery experiments to test circuit and behavioral hypotheses using a variety of manipulations, including gene overexpression, CRISPR/Cas9 gene editing, inducible technologies, optogenetic or DREADD cellular control, and cell type-specific expression.

The connections between genes, brain, and behavior are fundamental to our understanding of neurobiological processes. Songbird models meaningfully contribute to many fields, as the system has great strengths due to its developmental biology, quantifiable behavioral characterization, defined neural circuits for cognition and behavior, sequenced genome, and strong parallels to human speech acquisition^1^,^2^. However, likely due to immune system properties, viral gene delivery strategies commonplace in other systems have been more difficult to implement in songbird brain^3^,^4^. Viral infections in targeted brain areas can be functionally significant but efficiencies can be quite low and varied, and construct design can be impeded by limitations on the size of transgenes reliably packaged into viral particles^5^,^6^,^7^. Another approach for gene manipulation, the generation of transgenic songbirds, is possible but requires levels of investment not yet in-reach for most research projects^8^,^9^,^10^. We were therefore motivated to develop a reliable, efficient, and flexible strategy to manipulate the genome in brain cells of the songbird in service of directly testing gene-brain-behavior relationships.

We adapted *in vivo* electroporation procedures for use in the early Posthatch zebra finch chick. This procedure uses electrode paddles placed on the outside of the head to deliver DNA constructs into cells; the pulses disrupt plasma membranes and negatively-charged DNA is pulled towards the anode. To achieve genomic integration, we include the piggyBac transposase^11^,^12^. The piggyBac is a cut-and-paste transposase that recognizes inverted terminal repeat (ITR) sequences on the transposon to remove the cassette and integrate it into TTAA sites in the genome^13^,^14^,^15^. No obvious off-target effects have been reported. Notably, the piggyBac is expressed off its own plasmid, thus it can be used to integrate a variety of different transgene constructs, with the main requirement that they have the proper flanking ITR. The system affords many advantages, such as the co-electroporation of multiple constructs and temporally restricted integration into the genome^16^,^17^.

Here, we report a series of experiments to demonstrate that *in vivo* electroporation in Posthatch day 3 (P3) zebra finch chicks is an effective technique for long-term and neuroanatomically-restricted transgene expression. We targeted transgene expression to the auditory forebrain (AF) and show highly selective and stable localization of affected cells in this region up to P50. Comparison of CAG- and synapsin1-promoter-driven transgene expression demonstrates regulation in expected cell types. The procedure is effective with multiple constructs co-electroporated. This strategy is therefore appropriate for gene delivery experiments that test circuit and behavioral hypotheses using a variety of manipulations, including gene overexpression or interference with CRISPR editing, inducible technologies, optogenetic or DREADD cellular control, and cell type-specific expression.

## Methods

All procedures were conducted in accordance with the National Institute of Health guidelines for the care and use of animals for experimentation, and were approved by the University of Chicago Institutional Animal Care and Use Committee (ACUP #72220).

### Subjects

All chicks used in this study were hatched in laboratory breeding aviaries where birds were housed on a 14 h:10 h light:dark cycle, with seed and water provided *ad libitum*. Following electroporation, chicks were returned to their nests, where they received parental care until the end of the experiment. P5 was used for the purpose of assessing technical electroporation parameters. P30–50 were used to test the persistence (genomic integration) and neuroanatomical spread of FP+ cells in our targeted brain region through a critical portion of development that includes song learning^18^,^19^,^20^,^21^,^22^.

### Plasmids

**Figure 1:**
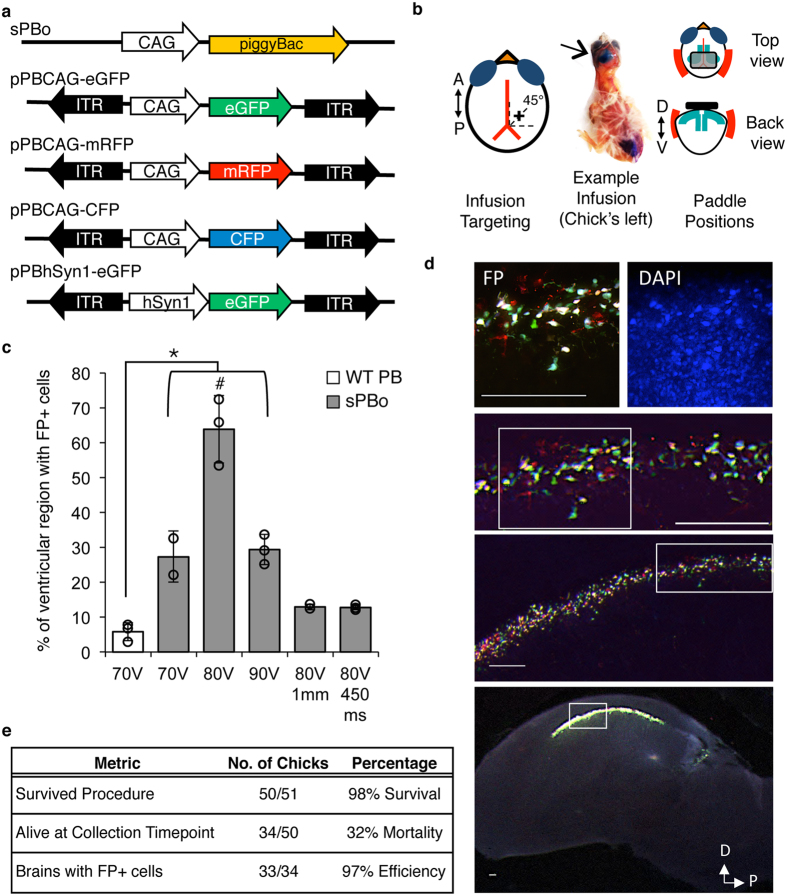
Hyperactive piggyBac transposase (sPBo) paired with 80 V pulses results in transgene expression in a large portion of the targeted area in zebra finch hatchlings. (**a**) Plasmid constructs used in this study. Donor pPB plasmids contain inverted terminal repeats (ITRs) surrounding the transgene expression cassettes, each containing a promoter (CAG or human Synapsin 1 [hSyn1] promoters) and fluorescent protein transgenes (eGFP, mRFP, or CFP). (**b**) Schematic of plasmid infusion targeting and paddle placements. Left panel: Infusions were targeted along a 45° angle visualized from the midline and Y_0_ (the anterior-most boundary of the cerebellum). Middle panel: Infusion into the chick’s left ventricle (arrow). Fast green dye in the plasmid mixture allows visual confirmation that the plasmid solution has filled the ventricle (arrow). Right panel: Schematic showing tri-electrode paddle positioning, as seen from the top and back views of the chick’s head. Positively-charged paddles (red) are placed on either side of the head, in line with the posterior-most portion of the filled lateral ventricles. The negatively-charged paddle is placed on top of the head, directly over the posterior-most portions of the lateral ventricles. (**c**) Quantitative comparison of the effects of various electroporation conditions on the percent of the targeted area containing fluorescent protein-positive (FP+) cells. sPBo is significantly more effective than wildtype (WT) piggyBac at all voltages tested (*p < 0.05; vs. 70, 80, 90 V sPBo). sPBo paired with 100 ms 80 V pulses delivered at an interpulse interval of 900 ms using 3 mm-wide paddles was significantly more efficient than all other conditions (^#^p < 0.001). n = 2–3 per group. Bar graphs indicate the group mean ± SEM; **○** = individual birds. (**d**) Representative sagittal plane images of fluorescent protein-positive (FP+) cells and DAPI-stained cell nuclei along the lateral ventricles 48 h after *in vivo* electroporation with sPBo, 3 mm-wide paddles, and 100 ms 80 V pulses delivered with an inter-pulse interval of 900 ms. White boxes outline areas magnified in the panels directly above. Scale bars = 100 μm. (**e**) Summary of the survival, mortality, and efficacy percentages obtained over the course of this study.

The hyperactive piggyBac construct (sPBo^23^,^24^ was obtained from Transposagen; Lexington, KY). The pPBCAG-eGFP, -mRFP, and -CFP plasmids, containing green, red, and cyan fluorescent proteins (FPs) flanked by piggyBac-specific ITRs^17^, as well as the wild-type (WT) piggyBac construct were generous gifts from the LoTurco laboratory (University of Connecticut). We constructed the pPBhSyn1-eGFP plasmid by first PCR-cloning the human synapsin1 promoter (hSyn1; 401 bp) from the pAAV-hSyn-DIO-hM3D-mCherry plasmid (Addgene plasmid #44361^25^) using the following primers: hSyn1 fwd, 5′-TAA GCA ACT AGT CTG CAG AGG GCC CTG CGT; hSyn1 rev, 5′-TGC TAA TCT AGA CGC CGC AGC GCA GAT GGT C. The hSyn1 promoter construct was then subcloned into the SpeI/XbaI sites of pPBCAG-eGFP. Plasmid schematics are depicted in Fig. 1a. For the CAG-only, triple FP electroporations, plasmid solutions contained all three fluorescent protein constructs and the sPBo at final concentrations of 500 μg/μl each in DNase-free water to test that multiple transgene constructs could be simultaneously integrated. For pPBhSyn1-eGFP + pPBCAG-mRFP electroporations, final plasmid concentrations were 500 μg/μl each. Results reported are for CAG-driven constructs unless otherwise stated.

### *In vivo* electroporation

P3 chicks were anesthetized with isoflurane. A small, ~3 mm, midline incision was made along the anterior-posterior axis of the scalp. The plasmid solution, containing 0.5% Fast-Green dye, was bilaterally injected via a pulled-glass capillary connected to a micropipette into the lateral ventricles (Fig. 1b). The dye enabled visualization and confirmation of intra-ventricular injection (Fig. 1b). 1–2 μl of solution was delivered into each ventricle, enough to fill the ventricular space. An ELP-01D cell and tissue electroporator (NPI Electronic; Tamm, Germany) connected to custom-shaped tri-electrode^26^ gold-plated paddles wetted with 0.1 M phosphate buffered saline (PBS) was used to deliver seven pulses (70, 80, or 90 V, each lasting 100 ms; see Fig. 1b for paddle placements). Pulses were delivered at an inter-pulse interval of either 900 or 450 ms, according to the specific experiment. After pulsing, chicks were immediately moved to a heating pad, and the incision was closed with Vetbond (3 M; St. Paul, Minnesota). Chicks were monitored until they resumed normal begging behaviors, after which they were returned to their home nests until the end of the experiment. The amount of time chicks were out of the nest for the entire procedure was 20–30 m. Chicks were monitored closely for the next 2–3 d to ensure that they were receiving parental care.

For all experiments, paddles were positioned to concentrate gene expression in the auditory forebrain (AF), a necessary brain region for sensory song learning and adult song recognition learning^22^,^27^,^28^.

### Tissue processing and imaging

At the end of experiments, brains were either drop-fixed or perfused, depending on the age of the chicks. P5 brains were removed and fixed in 4% paraformaldehyde (PFA) in 0.025 M PBS overnight at 4 °C. Juveniles aged P30–50 were intracardially perfused with 0.1 M PBS and 4% PFA, followed by dissection of the brains, which were stored in PFA overnight at 4 °C. All brains were then embedded in gelatin (8% in 0.1 M PBS) and fixed overnight at 4 °C. To cryoprotect the tissue, the gelatin-embedded brains were incubated overnight first in 15% and then 30% sucrose in 0.1 M PBS. Brains were serially sectioned at 50–55 μm with a cryostat. One series from each brain was DAPI-counterstained, mounted on Superfrost Plus slides (Fisher), dried overnight in the dark, and coverslipped with 2.5% polyvinyl alcohol (PVA) containing 0.5% 1,4-diazabicyclo[2.2.2]octane (DABCO).

Images were captured at the Integrated Light Microscopy Core Facility at the University of Chicago. Low-magnification widefield images were captured using the 2, 4, and 10X objectives of an Olympus IX81 inverted epifluorescence microscope with the Olympus Zero Drift Correction auto re-focusing system (Olympus Corporation of the Americas, Center Valley, PA) with a Hamamatsu Orca Flash 4.0 sCMOS camera (Hamamatsu Photonics, Skokie, IL). High magnification confocal images were captured with the 20X and 60X objectives on an Olympus DSU spinning disk confocal microscope (Olympus Corporation of the Americas, Center Valley, PA) with an Evolve EM-CCD camera (Photometrics, Tucson, AZ). Both microscopes were run by SlideBook v5.0 software (Intelligent Imaging Innovations, Denver, CO). Quantifications of fluorescence (area, density, localization) were conducted using thresholded - but otherwise unmanipulated - 16-bit greyscale images in FIJI^29^. For representative images, merged color and compressed Z-stack images were assembled with FIJI, and sharpness, brightness, and contrast were adjusted for optimal visual (publication) clarity.

### Proportion of area with fluorescent protein positive (FP+) cells at P5

We first used a 48-h timepoint to assess the level of transgene expression in cells surrounding the lateral ventricle, as the altricial zebra finch brain is largely unorganized at this age^30^,^31^. The density of FP+ cells in the subventricular region of the P5 brains is high, as demonstrated by DAPI staining (Fig. 1d). We therefore quantified the proportion of the subventricular area that contained cells expressing FPs as a measure of the initial efficacy of electroporation. On images captured with the 2X objective, we first selected the subventricular area with the FIJI brush tool, set to a diameter of 80 μm. Images were then thresholded to segment the fluorescent pixels from background. The resulting data represents the %Area FP+, which is a percentage of the total area selected by the brush tool that is occupied by FP+ cells. %Area FP+ measurements were averaged in all sections across both hemispheres for each individual for statistical analysis.

### Telencephalic cell counts and density calculations at P30, P40, P50

**Figure 2:**
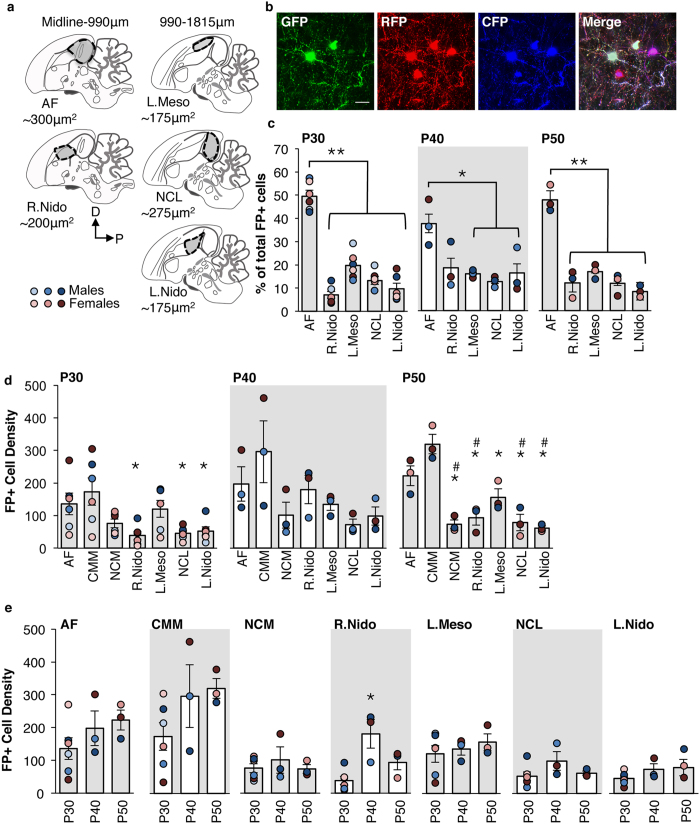
Proportion and density of FP+ cells across medial telencephalic brain regions. (**a**) Schematics of approximate boundaries and volumes of areas quantified for FP+ cells at P30, P40, P50. Redrawn and modified schematics are patterned on those in the ZEBrA Histological Atlas (www.zebrafinchatlas.org). (**b**) Representative images of GFP, RFP, and CFP+ cells in the P30 auditory forebrain (AF). Scale bar = 20 μm. (**c**) Percentage of the total number of FP+ cells located in the AF, the rostral nidopallium (R. Nido), the lateral mesopallium (L. Meso), the NCL, and the lateral nidopallium (L. Nido) at P30 (left graph), P40 (middle graph), and P50 (right graph). *p < 0.05 and **p < 0.001 between indicated groups. (**d** and **e**) Density of FP+ in the AF, caudomedial mesopallium (CMM), caudomedial nidopallium (NCM), R. Nido, L. Meso, NCL, and L. Nido at P30, P40, and P50. Data are graphed grouped either within age and across brain areas (**d**), or within brain area and across age (**e**). Bar graphs denote the mean number of FP+ cells per 500 μm^2^ in the indicated areas, with individual data points denoting each subject. (**d**) *p < 0.01, for all indicated groups as compared with CMM; ^#^*p* < 0.01 for all indicated groups as compared with AF. (**e**) *p < 0.01, as compared with the P30 group. Bar graphs denote the group mean ± SEM; **○** = individual birds. n = 6 (P30 age group), or 3 (P40 and P50 age groups).

We observed that while most FP+ cells were concentrated within AF, some FP+ cells were visible in the surrounding brain areas. To compare the density across defined neuroanatomical regions, we quantified FP+ cells in five areas in the medial telencephalon (Fig. 2a): AF, the rostral nidopallium, the lateral mesopallium, the caudolateral nidopallium (NCL), and the lateral nidopallium. We used the Histological Atlas on the Zebra Finch Expression Brain Atlas (ZEBrA, Oregon Health and Science University, Portland, OR; zebrafinchatlas.org) to guide the border definition of these five areas. The AF included both the primary auditory cortex analog, Field L, and the secondary auditory areas, the caudomedial mesopallium (CMM) and the caudomedial nidopallium (NCM), and spanned laterally from midline to 990 μm^28^. The rostral nidopallium covered the area from immediately anterior to CMM until immediately ventral to the visible anterior curl of the lateral ventricle. This area did not include the medial magnocellular nucleus of the anterior nidopallium (mMAN), and spanned laterally from midline to 990 μm. The remaining three areas, the lateral mesopallium, the NCL, and the lateral nidopallium spanned laterally from 990 to 1815 μm. The lateral mesopallium was defined as the area ventral to the lateral ventricle, and was ventrally and posteriorly defined by the mesopallium/nidopallium division. The NCL was defined as a semi-circular region bounded by the lateral ventricle and telencephalic border on the dorsal and posterior sides, excluding Field L, but including the medial-most portion of the arcopallium, as well as nucleus taeniae. The lateral nidopallium included the area anterior to Field L, and ventral to the lateral mesopallium area described above. The ventral border of the lateral nidopallium did not include the striatum, and the visible anterior end to the lateral ventricle was, as for lateral mesopallium, the anterior boundary.

FP+ cells in the P30, P40, and P50 brains were clearly distinguishable, allowing for quantification of individual FP+ cells. Using images captured with the 2X objective, we selected each of the five brain areas described above with the FIJI freehand selection tool, with FP+ cells segmented from the entire image using the Threshold function. The numbers of FP+ cells were then counted using the Analyze Particles command. From these numbers, we calculated the percent of the total number of FP+ cells found in each region. To normalize cell numbers to the area selected for inter-region density comparison, we divided the number of FP+ cells within each region by the area of that region. These density calculations were averaged across both hemispheres for each bird and brain area. Individual birds’ means for each brain area were used for statistical analysis.

### Plot profile analysis of FP+ cells across AF

**Figure 3:**
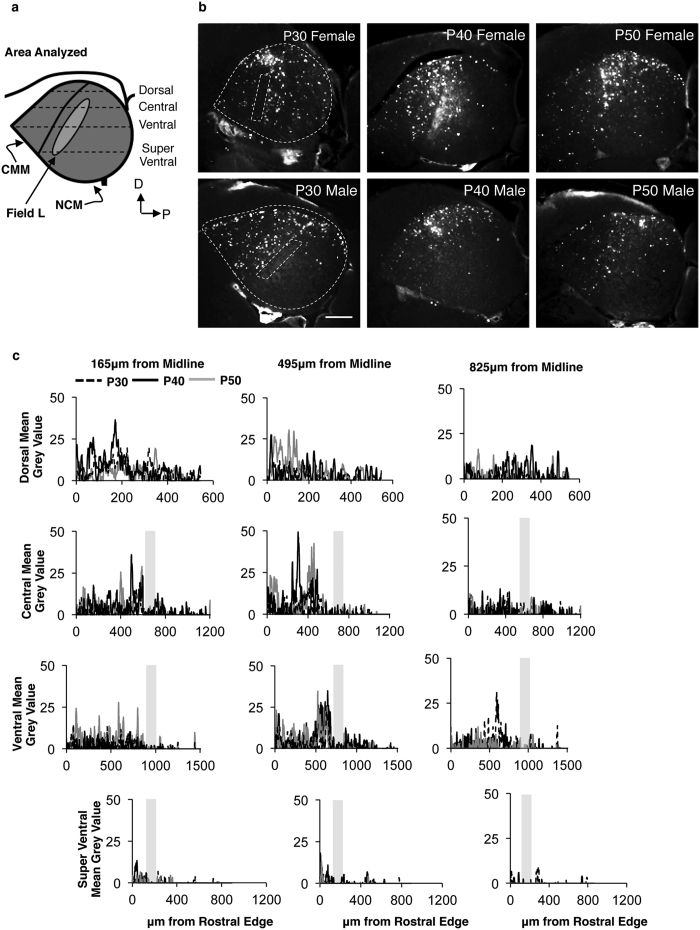
Distribution of FP+ cells throughout the AF at P30, P40, P50. (**a**) Schematic of AF and its subregions, CMM, NCM, and Field L. Dashed lines indicate the four rostral-caudal planes spanning the dorsal-ventral axis of AF used for Plot profile analysis: Dorsal, Central, Ventral, and Super Ventral. (**b**) Representative greyscale images of the AF in P30, P40, and P50 males and females. Contrast was adjusted for figure production only. Scale bar = 200 μm. (**c**) Plot profile analysis, quantifying the mean grey values along the four lines depicted in (**b**). Sections 165 μm (left column), 495 μm (middle column), and 825 μm (right column) from midline were analyzed. Central, Ventral, and Super Ventral lines included Field L, denoted with grey bars. Graphs are centered around Field L for visual comparison. Note that x-axes vary along the dorsal-ventral axis, reflecting the change in the anatomical length of AF. Lines represent the age group mean. n = 4 (P30), and 3 (P40 and P50).

To analyze the distribution of cells across AF at P30, P40, and P50, we performed Plot Profile analysis of line selections on four rostral-caudal planes in thresholded 4X images. This analysis quantifies the grey values (corresponding to the intensity of the fluorescence) in 1 μm bins across the length of each plane. Summation of the grey values for every 1 μm bin therefore results in a measure of the total amount of fluorescence across that rostral-caudal plane. In the dorsal half of AF, we quantified FP+ cells along three evenly-distributed rostral-caudal planes (~85 μm apart) across the dorsal-ventral axis of AF (Dorsal, Central, and Ventral planes; Fig. 3a). In addition, we also analyzed FP+ cells along one rostral-caudal plane centered in the ventral half of AF (Super Ventral; Fig. 3a), ~420 μm ventral to the ventral plane. For this analysis, we used brain sections 165, 495, and 825 μm from midline. Plot profile analyses of each plane and section were averaged across both hemispheres for an individual bird mean. Individual bird means were averaged to obtain age group means and generate line plots. These line plots were constructed to visually compare both the rostral-caudal and dorsal-ventral localization of fluorescence, and thus FP+ cells. To compare the total amount of fluorescence, individual bird means of the summed gray values were used.

### NeuN+ immunohistochemistry and FP+ cellular colocalization analysis

To determine the proportion of FP+ cells that were neurons in P30, P40, and P50 AF, we conducted immunohistochemistry for the neuron-specific marker, NeuN, and quantified FP+ and NeuN+ cells within four subregions of the dorsal half of AF (below). Sections were permeabilized with 0.1 M PBS containing 0.03% Triton-X. After three, 10 m washes with 0.1 M PBS containing 0.5% Tween-20 (PBST), sections were blocked for 1 h at room temperature in 3% normal horse serum (NHS; #S2000 Vector Labs, Burlingame, CA, USA). A monoclonal mouse IgG anti-NeuN primary antibody (1:10,000 in 1% NHS; #MAB377, EMD Millipore, Billerica, MA) was applied to the sections for a 2 h incubation at room temperature. Sections were then washed and incubated in Dylight 649 Horse anti-goat IgG secondary (1:500; #DI2649, Vector Labs) for 2 h at room temperature. Sections were counterstained with DAPI for nuclear identification, mounted on Superfrost Plus slides (Fisher), dried overnight in the dark, and coverslipped with 2.5% PVA containing 0.5% DABCO.

**Figure 4:**
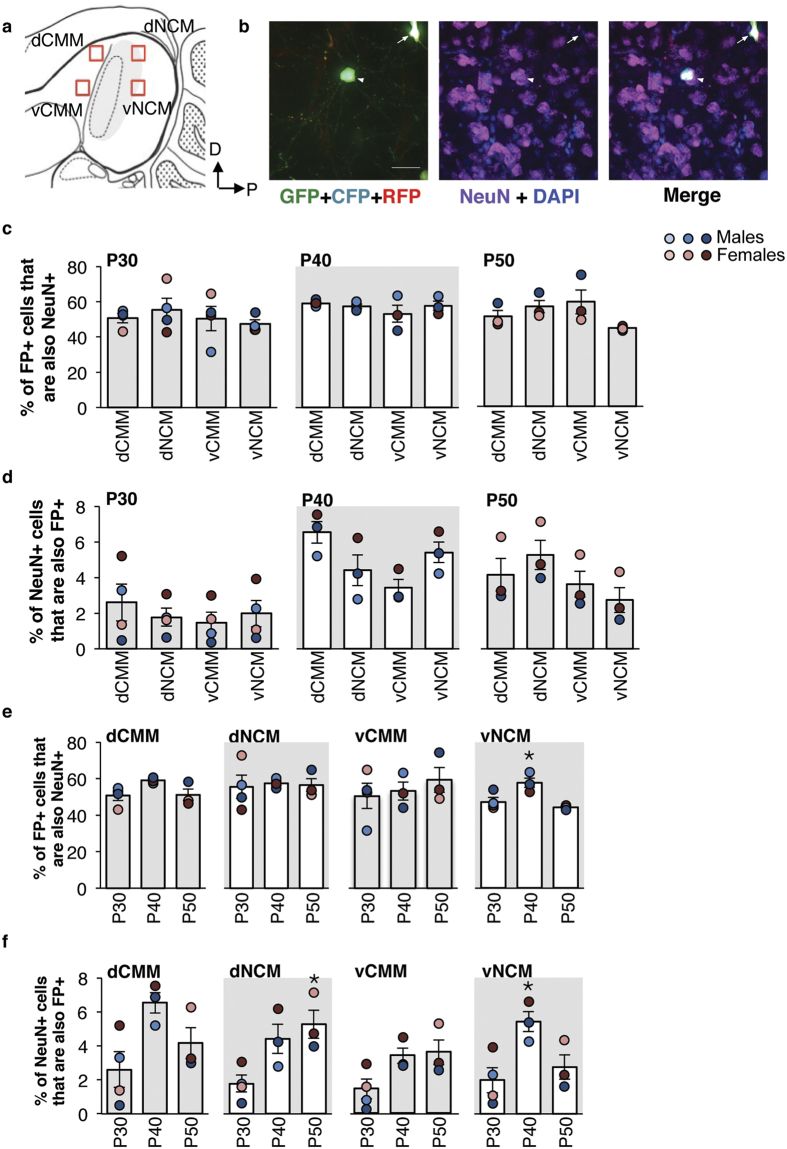
Colocalization of fluorescent proteins and NeuN. (**a**) Schematic of the areas imaged for the dCMM, dNCM, vCMM, and vCMM quantifications. (**b**) Example image of the visual confirmation of FP+ cells that are also NeuN-positive (NeuN+; arrowhead), and FP+ cells that are NeuN-negative (arrow). Scale bars = 25 μm. (**c** and **e**) Proportion of FP+ cells that are also NeuN+. *p < 0.01, as compared with the P30 and P50 age groups within vNCM. (**d** and **f**) Proportion of NeuN+ cells that were also FP+. *p < 0.01, as compared with the area-matched P30 age group. Data are presented as the proportion of co-labeled cells within age and across brain area (**c** and **d**) or within brain area and across age (**e** and **f**). Bar graphs represent the group mean ± SEM; **○** = individual birds. n = 4 (P30), and 3 (P40 and P50).

We obtained 20X images (409.6 × 409.6 μm) from along the dorsal boundary of both the CMM and the NCM (dCMM and dNCM), and ~450 μm ventral to the dorsal boundary (vCMM and vNCM; Fig. 4a). To quantify the number of FP+ cells that were also NeuN+, we marked each FP+ cell with the multipoint selection tool (FIJI), then manually counted the overlap in the NeuN image channel. For the converse, the percent of NeuN+ cells that were also FP+, we thresholded the NeuN channel to get the total number of cells with Analyze Particles (FIJI), and divided the number of co-expressing cells by this total. We quantified FP+ and NeuN+ cellular colocalization for the pPBhSyn1-eGFP+ pPBCAG-mRFP experiment in the same way. DAPI-stained nuclei counts were conducted as described for acquisition of total NeuN+ cell counts. Cell counts were obtained for sections from midline to 495 μm lateral in both hemispheres^32^. The data for each bird were combined and averaged to obtain an individual bird mean for statistical analysis.

### Statistics

Significant effects between Pulsing parameters, Age groups, Brain areas, or Subregions were assessed using one-way Analysis of Variance (ANOVA), while significant main effects and interactions between Sex and Brain area, Promoter and Age, or Promoter and Subregion were ascertained using a two-way ANOVA using StatPlus software (α < 0.05; AnalystSoft, Walnut, CA, USA). *Post-hoc* comparisons (Tukey’s HSD when sample groups were of the same size, and Tukey-Kramer in the event of unequal sample sizes) were conducted to determine the source of variation if main effects and/or interactions were obtained. For the plot profile analysis, mixed model ANOVA (XLStat, Addinsoft, New York, NY) was used to determine the source of variation, with individual birds as the random factor, and Age, Plane, and Section as the fixed factors.

## Results

### High survival and efficacy of *in vivo* electroporation procedure in zebra finch chicks

In this study, we electroporated a total of 51 chicks. Of those, 50 chicks survived the procedure, a survival rate of 98%. Out of the 50 chicks that survived the electroporation procedure, 34 were still alive at their assigned collection timepoint (P5, P30, P40, or P50). This reflects a survival rate of 68%, comparable to that previously reported in unmanipulated laboratory breeding conditions^33^. Furthermore, 33 of the 34 remaining chicks’ brains contained FP+ cells, an efficacy rate of 97%.

### *In vivo* electroporation effectively introduces transgenes into zebra finch brain

We considered pulsing voltage and frequency, as well as the capacity for transgene genomic integration, as major factors that affect the number of FP+ cells. We used a triple-electrode probe to produce bilateral FP+ cells from minimal exposure to the electrical pulses, and to gain better control over regional targeting^26^. One-way ANOVA was conducted to ascertain the effect of each Pulsing parameter (piggyBac variant, voltage, paddle size, and interpulse interval) on the production of FP+ cells 48 h post-electroporation (Fig. 1c). There was a significant effect of Pulsing parameter on the %Area FP+ (Table 1). Using 70 V pulses, we found that the use of the hyperactive sPBo piggyBac transposase resulted in a significantly greater %Area FP+, as compared with WT piggyBac (*p* = 0.015). Using the sPBo piggyBac, 80 V pulses resulted in a significantly greater %Area FP+ as compared with 70 V or 90 V pulses (*p* = 0.0003 and 0.0005, respectively). With 80 V pulses, decreasing the size of the paddles (from 3 mm to 1 mm, in an effort to increase targeting resolution), or the interpulse interval (from 900 ms to 450 ms) both significantly lowered the %Area FP+ (*p* = 0.0001, and 0.0001, respectively). Since the largest %Area FP+ was obtained after a combined use of the sPBo piggyBac, 100 ms 80 V pulses delivered 900 ms apart, and 3 mm-wide paddles, we used these pulsing conditions for the remainder of the study. We observed densely-packed FP+ cells in the subventricular area without the need for signal enhancement via immunohistochemistry (Fig. 1d). Moreover, under higher magnification, the FP+ cells presented a wide range of color and fluorescence intensity, indicating stochastic expression of the three electroporated FP constructs (red, green, and cyan; Fig. 1d, middle and top panels).

### AF contains higher proportion of FP+ cells than surrounding telencephalon at P30, P40, and P50

We first asked if our method preferentially targeted cells populating AF, the region we targeted. Since there was no effect of Sex (F_(1,28)_ = 0.06, *p* = 0.8) within our initial P30 group that included 3 males and 3 females, we used a combination of males and females for all additional P30, P40, P50 experiments and analysis. For all three ages tested, AF contained a significantly greater proportion FP+ cells than the four other telencephalic regions (Table 2). At P30 and P50, AF contained 49.6 and 49.4% of the total number of FP+ cells, respectively (Fig. 2b). There was a significant effect of Brain area for both the P30 and P50 birds. *Post hoc* comparisons revealed a significantly greater proportion of the FP+ cells located in AF as compared with the rostral nidopallium (P30: *p* = 0.0001; P50: *p* = 0.0002) the lateral mesopallium (P30: *p* = 0.0001; P50: *p* = 0.0002), the lateral nidopallium (P30: *p* = 0.0001; P50: *p* = 0.0002), and the NCL (P30: *p* = 0.0001; P50: *p* = 0.0002). At P40, there was a significant effect of brain area, and 37.2% of the total number of FP+ cells were located in AF (Table 2 and Fig. 2b). *Post hoc* comparisons within P40 revealed a significantly greater proportion of FP+ cells in AF than in the lateral mesopallium (*p* = 0.04), the lateral nidopallium (*p* = 0.04), and the NCL (*p* = 0.018). Although a smaller proportion of total FP+ cells are localized to the P40 AF, there are no significant differences in the total number of FP+ cells in the P30, P40, and P50 AFs (F_(2,9)_ = 1.25, *p* = 0.33).

### Within the P30 and P50 AF, the density of FP+ cells is highest in the CMM

Initially, we used a two-way ANOVA to test for significant main effects or interactions between Age and Brain area on the FP+ cell density. Since we found significant main effects of both factors (Age: F_(2,63)_ = 8.9, *p* = 0.00004; Brain area: F_(6,63)_ = 13.4, *p* = 9.9e-10), but no interaction (F_(12,83)_ = 1.14, *p* = 0.35), we proceeded to analyze within these two factors.

At all ages, CMM had the highest density of FP+ cells; we used one-way ANOVAs to further determine the effect of Brain area on FP+ cell density at each age (Fig. 2c). At P30, we found a significant effect of Brain area (Table 3), and *post hoc* comparisons revealed significant differences between CMM and the NCL (*p* = 0.018), the lateral nidopallium (*p* = 0.011), and the rostral nidopallium (*p* = 0.006). We did not find a significant effect of Brain area at P40 (Table 3) although CMM tended to have higher FP+ cell densities than the other telencephalic regions. At P50, there was a significant main effect of Brain area (Table 3). *Post hoc* comparisons revealed significant differences between the AF (including Field L) and the NCL (*p* = 0.004), the lateral nidopallium (*p* = 0.01), NCM (*p* = 0.007), and the rostral nidopallium (p = 0.02). *Post hoc* analysis also revealed significant differences between CMM and the lateral mesopallium (*p* = 0.003), NCL (*p* = 0.0001), the lateral nidopallium (*p* = 0.0001), NCM (*p* = 0.001), and the rostral nidopallium (*p* = 0.006).

### There is little effect of age on the FP+ cell density in the P30, P40, and P50 telencephalon

We conducted one-way ANOVAs to ascertain the effect of Age on the density of FP+ cells within each of the telencephalic areas (Fig. 2d). There was no effect of Age in any brain area except rostral nidopallium (Table 3), where *post hoc* Tukey-Kramer revealed a significant difference between the P30 and P40 age groups (*p* = 0.005).

### Plot profile analysis demonstrates dorsal-ventral and anterior-posterior FP+ cell gradients within AF

Because of the AF-biased localization of FP+ cells, we used plot profile analysis to assess their distribution within its boundaries. Consistent with our observation that few FP+ cells populated the primary auditory cortex, Field L (Fig. 3b), very low gray values were obtained for the areas spanning Field L (Fig. 3c). Plot profile analysis visually suggests greater overall mean grey values in the more dorsal versus ventral aspects of AF, as well as in the rostral versus caudal portions of AF (Fig. 3c). This latter point emphasizes the CMM-weighted concentration of FP+ cells (Fig. 2c). To better quantify the dorsal-ventral distribution observations, we used next used the sum total of grey values to compare the total level of fluorescence across each of the planes measured. We used a mixed model ANOVA, using individual birds as the random factor, to test the effects of the following fixed factors: Plane (Dorsal, Central, Ventral, and Super Ventral) and Age (P30, P40, and P50), Section (165, 495, or 825 μm) and Age, and Plane and Section. We found significant main effects of both Plane (F_(3,105)_ = 12.7, *p* = 0.0001) and Section (F_(2,105)_ = 7.38, *p* = 0.001), but not of Age (F_(2,105)_ = 0.18, *p* = 0.84). The gradient of FP+ cells was therefore weighted towards the more dorsal planes of the two medial sections at P30, P40, and P50.

### Neuronal expression of FP transgenes in the AF

We analyzed the proportion of FP+ cells that were neurons, and the proportion of neurons that were FP+ within four AF subregions: dCMM, vCMM, dNCM, and vNCM (Fig. 4b). First, we compared the total number of FP+ cells, NeuN+ cells (neurons), and DAPI-stained nuclei (all cells) in P30, P40 and P50 dCMM, vCMM, dNCM, and vNCM. When compared within each age, there were no significant effects of Subregion on the total number of FP+ cells, total number of NeuN+ cells, or DAPI-stained nuclei (Table S1). Moreover, within each of the four AF subregions, we did not find a significant effect of Age on the number of FP+ cells, NeuN+ cells, or DAPI-stained nuclei (Table S2). Thus, the number of FP+ cells, neurons, and total cells are stable across both AF subregion and age.

We next tested if the colocalization of FPs and NeuN differs across AF subregion. Approximately half of all FP+ cells across each of the four AF subregions in the three age groups were also NeuN+ (Fig. 4c). There was no significant effect of Subregion on the proportion of FP+ cells that were also NeuN+ at P30, P40, or P50 (Table 4 and Fig. 4c). Similarly, there was no significant effect of Subregion on the proportion of NeuN+ cells that were also FP+ at P30, P40, and P50 (Table 4 and Fig. 4d).

When we tested for an effect of age on the proportion of FP+ cells that were NeuN+ (Fig. 4e), we found a significant effect of Age in the vNCM (Table 4). *Post hoc* Tukey-Kramer revealed significant differences between the P40 age group and both the P30 (*p* = 0.03) and P50 (*p* = 0.01) age groups. There was no significant effect of Age in the dCMM, the dNCM, or the vCMM (Table 4). Thus, in vNCM, proportions of NeuN-expressing FP+ cells and FP-expressing NeuN+ cells tend to increase across age.

For the proportion of NeuN+ cells that were also FP+, there was a significant effect of Age in the dNCM (Table 4 and Fig. 4f). *Post hoc* Tukey-Kramer revealed significant differences between the P30 and P50 age groups (*p* = 0.04). There was also a significant effect of Age in the vNCM (Table 4). *Post hoc* Tukey-Kramer revealed significant differences between the P30 and P40 age groups (*p* = 0.03). There were no significant effects of Age in either the dCMM, or the vCMM (Table 4).

### The hSyn1 promoter enhances the proportion of transgene-expressing cells that are neurons

**Figure 5:**
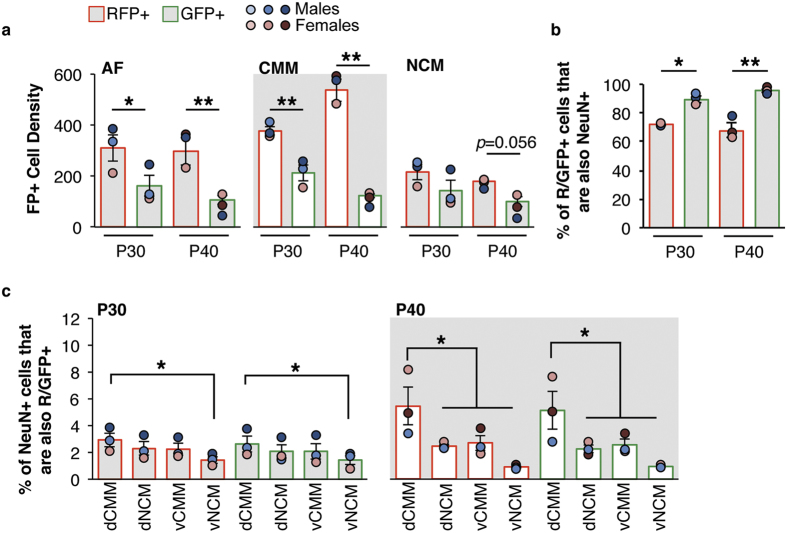
Synapsin promoter enhances neuronal expression of fluorescent proteins. The hSyn1 promoter was used to drive eGFP expression and CAG was used to drive universal transcription of RFP. (**a**) Density of either RFP-positive (RFP+) or eGFP-positive (GFP+) in AF (left graph), CMM (middle graph), or NCM (right graph) at P30 and P40. *p < 0.05 and **p < 0.001, as compared with age-matched RFP+. (**b**) Proportion of RFP+ or GFP+ cells that are also NeuN+. Data are presented as the mean across the dCMM, vCMM, dNCM, and vNCM subregions of AF. *p < 0.05 between indicated groups. (**c**) Proportion of NeuN+ cells that are also either RFP+ or GFP+ in each AF subregion at P30 (left graph) and P40 (right graph). *p < 0.05 between indicated groups. All data are presented as the group mean ± SEM; **○=**individual birds. n = 3 per age.

We tested if a neuron-specific promoter could be used to increase the cellular specificity of electroporated transgene expression. To do so, we co-electroporated the pPBhSyn1-eGFP and pPBCAG-mRFP plasmids. First, we compared the effectiveness of the CAG and hSyn1 promoters. We used a two-way ANOVA to test the effects of Promoter (CAG or hSyn1) and Age (P30 or P40) on the density of GFP+ or RFP+ cells in CMM, NCM, and AF as a whole (Fig. 5a). In CMM, there was a significant main effect of Promoter, but not of Age on the FP+ cell density, and there was a Promoter × Age interaction (Table 5). In CMM, RFP+ cell density was higher than GFP+ cell density (P30: *p* = 0.002; P40: *p* = 0.0002). In NCM, there was a significant main effect of Promoter, but not of Age in the FP+ cell density, and no Promoter × Age interaction (Table 5). In P30 NCM, RFP+ cell density was not different than GFP+ cell density (*p* = 0.11). However, in P40 NCM, RFP+ cell density tended to be higher than GFP+ cell density (*p* = 0.056). In AF, there was a significant main effect of Promoter, but not of Age, and no Promoter × Age interaction on the FP+ cell density (Table 5). As expected, AF RFP+ cell density (CAG-driven) was higher than GFP+ cell density (hSyn1-driven; P30: *p* = 0.03; P40: *p* = 0.004). The technical efficiencies when we electroporated with pPBhSyn1-eGFP and pPBCAG-mRFP or with three CAG-driven FP genes were equivalent. We found no statistical difference between the density of CAG-driven RFP+ cells in AF for this experiment and the density of FP+ cells obtained in AF for the CAG-only experiment above (F_(4,7)_ = 2.45, *p* = 0.14).

We next analyzed for cellular colocalization of FPs and NeuN in dCMM, vCMM, dNCM, and vNCM. In all AF subregions for both P30 and P40 age groups, a greater proportion of GFP+ population of cells were also NeuN+, as compared with the RFP+ population. We therefore averaged measurements across AF Subregions to obtain an overall measure of co-localization in AF, and tested for any effects of Age and/or Promoter (Fig. 5b). There was a significant effect of Promoter, but not of Age, and no interaction (Table 5). In both P30 and P40 age groups there was a significantly higher level of GFP-NeuN co-localization than RFP-NeuN (P30: *p* = 0.002; P40: *p* = 0.0003).

Finally, we quantified the proportion of NeuN+ cells that were either RFP+ or GFP+ (Fig. 5c). There was a significant main effect of Subregion at both P30 and P40 (Table 6). *Post hoc* analysis revealed a significant difference between the dCMM and the vNCM (*p* = 0.02) at P30, and dCMM and all of the other areas (dNCM: *p* = 0.0005; vCMM: *p* = 0.002; vNCM: *p* = 0.00001) at P40. We found no significant effects of Promoter or Subregion × Promoter interaction at P30 or at P40 (Table 6).

## Discussion

We took advantage of a method for gene delivery, *in vivo* electroporation, to express exogenous DNA constructs in the developing zebra finch brain. Our survivability rate (98%) compares favorably to that obtained for rodents (70–100%); to our knowledge, survivability rates have not been explicitly reported in other birds^34^,^35^,^36^. The number of electroporated zebra finch chicks possessing cells expressing FPs (97%) matches, and sometimes exceeds, those achieved for both rodent embryos (70–90%) and developing chickens (<90%)^16^,^34^,^36^. We used multiple methods to assess FP-expressing cells spanning different ages throughout zebra finch development. In zebra finches, the percentage of cells expressing FPs (50–70%) is comparable to that reported for developing chickens (50–85%), and exceeds that reported for rodents (25%)^36^,^37^,^38^. Moreover, the relatively low inter-individual variability in transgene expression is a marked improvement over viral-mediated gene transfer, which can result in ~10-fold differences in the density of transgene-expressing cells between experiments^5^,^6^. Thus, on several metrics the efficiency of the method in zebra finches compares favorably to other systems in which substantial advances have been made.

Juvenile male zebra finches copy song from a “tutor” bird during a developmental sensitive period that runs P30–65; maximal levels of song copying can be achieved by tutor experiences from P40–50^18^,^19^,^20^,^22^. In poultry, transgene expression has been reported to last for only 11 days post-electroporation, although in some rodent experiments, transgene expression was measurable for up to least 6 weeks^39^,^40^. To determine if our P3 electroporation strategy is suitable for genomic manipulation combined with tutor experience, we quantified FP+ cells at P30, P40, and P50. We observed FP+ cells at P30, P40, and P50, with no difference in the total number or within-AF distribution of FP+ cells at those ages. This is indicative of successful genomic integration. It also demonstrates cells born at or around P3 likely survive through P50. It is possible that different cells could be targeted if electroporation was performed at a different developmental age, affecting a distinct set of stem cells. FP+ cells occupy approximately 40% of the area of AF; pharmacological molecular disruption of similarly-sized region of AF is sufficient to prevent normal levels of tutor song copying^22^. This suggests that the current parameters can be combined with measures of song learning to probe functional gene-brain-behavior relationships.

Nearly half of all telencephalic FP+ cells, and at least twice as many as other adjacent regions, were localized within AF. Within AF, cells are preferentially found in the secondary auditory cortex analogs, NCM and CMM, relative to primary auditory cortex Field L. Statistically, there were few differences between NCM and CMM cell density, but plot profile analysis illustrates a greater number of FP+ cells are in CMM compared to NCM. We also detected a decreasing gradient of FP+ cell number from dorsal to ventral. The dorsal bias may reflect patterns of migration; newly divided cells are still born at P30 in the zebra finch and we cannot distinguish how old our FP+ cells are^41^,^42^. The concentration of FP+ cells in CMM as compared with NCM can be leveraged in the context of the functional difference between these two regions. For example, it has been proposed that NCM is disproportionately involved in processing song novelty whereas CMM evaluates the social significance of song; targeted gene manipulation could test underlying mechanisms of these differences^43^.

Across experiments, approximately 60% of the FP+ cells were also NeuN+ when the CAG promoter was used. The proportion of NeuN+ cells compared to total cells is closer to 40%, indicating that CAG-driven transcription is enriched in neurons. We can obtain nearly exclusive (~92%) neuronal expression using the neuron-specific hSyn1 promoter. Despite the large area of AF that contains cells expressing transgenes, the number of neurons that also express FP are below 10% regardless of CAG- or hSyn1-driven transcription. Thus, the universal CAG promoter targets the same proportion of neurons as the hSyn1 promoter, but also expresses transgene in other cell types. Since the songbird brain contains a very high number of neurons, at densities exceeding those of mammalian brains, it is conceivable that our current electroporation parameters already target a maximal number of neuronal cells, regardless of the promoter used and the hSyn1 promoter drives expression exclusively in neurons^44^. It is also possible that electroporation at a different age will target a different set of progenitors that give rise to a different complement of cell types.

We demonstrate that posthatch *in vivo* electroporation is a rapid and straightforward method to reliably deliver gene expression constructs to a behaviorally-relevant brain area, AF. Our demonstration of universal and neuron-enhanced overexpression is a proof of concept that opens up multiple possibilities for probing gene-brain-behavior interdependencies. Due to the flexibility and ease of plasmid design and construction, neurobiological processes uniquely modeled by the zebra finch may now be probed with greater efficiency and precision. For example, simple ligation of gene interference constructs from validated, commercially available plasmids (such as RNAi and CRISPR/Cas9) into the piggyBac-ITR backbone can be conducted to expeditiously test how age and experience-dependent gene expression defines and supports learning^45^. In the same way, levels of cell activity may be controlled and manipulated with DREADD and optogenetic tools to identify the cellular networks responsible for aspects of song learning^46^,^47^. Overexpression of proteins with point mutations can be used to functionally connect signaling cascades to the cellular processes that encode experience and mediate learning. In addition, inducible promoter systems can be combined with any of the above approaches to add a temporal dimension to the experimental manipulation^48^. The range of color and intensity of fluorescence observed is indicative of simultaneous integration of multiple constructs in each cell. Integration of multiple constructs can be advantageous when investigating complex signaling cascades. Although we focused on producing transgene-expressing cells in AF, it is possible to adjust paddle placements to manipulate gene expression in other brain areas of interest. This highly reliable technique is therefore a readily-accessible, cost-effective, flexible and rapid method for gene manipulation in a neuroethological model of learned vocal communication, social behavior, brain development, and ecology and evolution.

## Additional Information

**How to cite this article**: Ahmadiantehrani, S. and London, S. E. A reliable and flexible gene manipulation strategy in Posthatch zebra finch brain. *Sci. Rep.* **7**, 43244; doi: 10.1038/srep43244 (2017).

**Publisher's note:** Springer Nature remains neutral with regard to jurisdictional claims in published maps and institutional affiliations.

## Supplementary Material

Supplementary Tables

## Figures and Tables

**Table 1:** Optimization of electroporation parameters.

Pulsing Parameter	Mean	SEM	Main effect	P-value
WT piggyBac, 70 V	5.8	2.58	F_(5,11)_ = 49.45	3.60E-07
sPBo, 70 V	27.34	7.32		
sPBo, 80 V	63.85	9.66		
sPBo, 90 V	29.39	4.32		
sPBo, 80 V, 1 mm paddles	12.87	0.8		
sPBo, 80 V, 450 ms interpulse interval	12.82	0.67		

**Table 2:** Telencephalic distribution of FP+ cells.

Age	Telecephalic Region	Mean	SEM	Main effect	P-value
P30	AF	49.59	2.49	F_(4,25)_ = 68.01	4.56E-13
	R. Nido	7.01	1.49		
	L. Meso	19.81	2.34		
	NCL	13.28	1.70		
	L. Nido	9.76	2.27		
P40	AF	37.21	3.98	F_(4,10)_ = 4.98	1.80E-02
	R. Nido	18.30	4.06		
	L. Meso	15.82	0.68		
	NCL	12.48	0.91		
	L. Nido	16.18	3.87		
P50	AF	49.37	2.42	F_(4,10)_ = 38.2	4.98E-06
	R. Nido	12.37	2.43		
	L. Meso	17.46	1.29		
	NCL	12.16	1.96		
	L. Nido	8.64	1.00		

**Table 3:** Summary of FP+ cell density.

	Age	Brain Area	Mean	SEM	main effect	P-value
FP+ Cell Density Within Age	P30	AF	135.36	33.12	F_(6,35)_ = 4.58	2.00E-03
		CMM	173.53	42.06		
		NCM	75.94	13.41		
		R. Nido	38.93	12.41		
		L. Meso	120.40	25.48		
		NCL	46.23	7.87		
		L. Nido	52.68	13.43		
	P40	AF	197.46	53.05	F_(6,14)_ = 2.45	0.08
		CMM	295.93	95.48		
		NCM	101.34	40.36		
		R. Nido	180.30	43.75		
		L. Meso	134.72	18.47		
		NCL	72.71	17.27		
		L. Nido	98.66	28.17		
	P50	AF	222.44	30.33	F_(6,14)_ = 16.8	1.00E-05
		CMM	319.36	29.80		
		NCM	73.62	13.38		
		R. Nido	93.79	23.16		
		L. Meso	156.00	25.87		
		NCL	78.61	25.02		
		L. Nido	61.84	6.27		
		
	**Brain Area**	**Age**	**Mean**	**SEM**	**main effect**	**P-value**
FP+ Cell Density Within Brain Area	AF	P30	135.36	33.12	F_(2,9)_ = 1.43	0.28
		P40	197.46	53.05		
		P50	222.44	30.33		
	CMM	P30	173.53	42.06	F_(2,9)_ = 2.18	0.17
		P40	295.93	95.48		
		P50	319.36	29.80		
	NCM	P30	75.94	13.41	F_(2,9)_ = 0.43	0.66
		P40	101.34	40.36		
		P50	73.62	13.38		
	R. Nido	P30	38.93	12.41	F_(2,9)_ = 9.34	0.006
		P40	180.30	43.75		
		P50	93.79	23.16		
	L. Meso	P30	120.40	25.48	F_(2,9)_ = 0.45	0.65
		P40	134.72	18.47		
		P50	156.00	25.87		
	NCL	P30	46.23	7.87	F_(2,9)_ = 1.87	0.21
		P40	72.71	17.27		
		P50	78.61	25.02		
	L. Nido	P30	52.68	13.43	F_(2,9)_ = 1.61	0.25
		P40	98.66	28.17		
		P50	61.84	6.27		

**Table 4:** Colocalization of FPs and NeuN.

	Age	AF Subregion	Mean	SEM	main effect (Subregion)	P-value
% of FP+ cells that are also NeuN+	P30	dCMM	50.69	2.63	F_(3,12)_ = 0.44	0.73
		dNCM	55.48	6.45		
		vCMM	50.57	6.96		
		vNCM	47.33	2.32		
	P40	dCMM	59.10	0.88	F_(3,8)_ = 0.59	0.64
		dNCM	57.36	1.28		
		vCMM	53.19	4.88		
		vNCM	57.81	2.65		
	P50	dCMM	51.08	3.19	F_(3,8)_ = 1.86	0.21
		dNCM	56.57	3.61		
		vCMM	59.28	6.75		
		vNCM	44.40	0.53		
% of NeuN+ cells that are also FP+	P30	dCMM	2.60	1.05	F_(3,12)_ = 0.41	0.75
		dNCM	1.77	0.50		
		vCMM	1.47	0.58		
		vNCM	1.98	0.74		
	P40	dCMM	6.53	0.60	F_(3,8)_ = 3.26	0.08
		dNCM	4.41	0.86		
		vCMM	3.42	0.45		
		vNCM	5.41	0.58		
	P50	dCMM	4.17	0.92	F_(3,8)_ = 1.31	0.33
		dNCM	5.27	0.83		
		vCMM	3.63	0.73		
		vNCM	2.74	0.71		
% of FP+ cells that are also NeuN+	dCMM	P30	50.69	2.63	F_(2,7)_ = 2.93	0.12
		P40	59.10	0.88		
		P50	51.08	3.19		
	dNCM	P30	55.48	6.45	F_(2,7)_ = 0.04	0.97
		P40	57.36	1.28		
		P50	56.57	3.61		
	vCMM	P30	50.57	6.96	F_(2,7)_ = 0.41	0.68
		P40	53.19	4.88		
		P50	59.28	6.75		
	vNCM	P30	47.33	2.32	F_(2,7)_ = 8.63	0.013
		P40	57.81	2.65		
		P50	44.40	0.53		
% of NeuN+ cells that are also FP+	dCMM	P30	2.60	1.05	F_(2,7)_ = 4.08	0.07
		P40	6.53	0.60		
		P50	4.17	0.92		
	dNCM	P30	1.77	0.50	F_(2,7)_ = 5.75	0.033
		P40	4.41	0.86		
		P50	5.27	0.83		
	vCMM	P30	1.47	0.58	F_(2,7)_ = 3.57	0.09
		P40	3.42	0.45		
		P50	3.63	0.73		
	vNCM	P30	1.98	0.74	F_(2,7)_ = 5.63	0.034
		P40	5.41	0.58		
		P50	2.74	0.71		

**Table 5:**
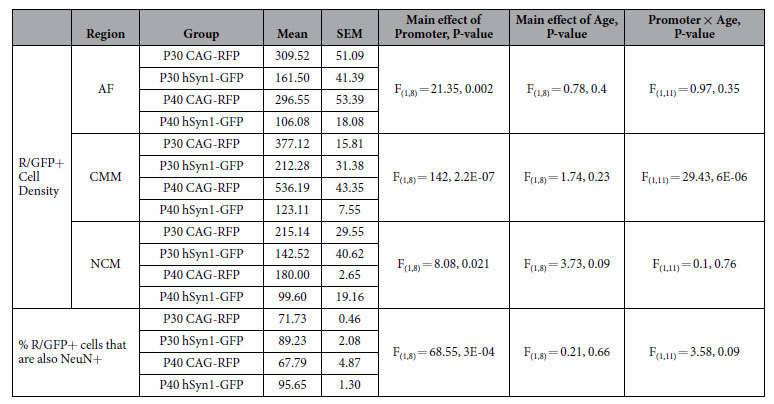
Summary of CAG- and hSyn1-driven FP expression.

**Table 6:** Proportion of neurons containing FPs.

	Age	Promoter	Subregion	Mean	SEM	Main effect of Subregion, P-value	Main effect of Age, P-value	Promoter × Age, P-value
% of NeuN+ cells that are also R/GFP+	P30	CAG (RFP)	dCMM	2.93	0.51	F_(2,24)_ = 3.32, 0.04	F_(3,24)_ = 0.32, 0.72	F_(6,35)_ = 0.03, 0.99
			dNCM	2.30	0.50			
			vCMM	2.23	0.45			
			vNCM	1.43	0.25			
		hSyn1 (GFP)	dCMM	2.63	0.59			
			dNCM	2.08	0.50			
			vCMM	2.08	0.58			
			vNCM	1.43	0.33			
	P40	CAG (RFP)	dCMM	6.80	1.75	F_(3,24)_ = 16.8, 4.4E-06	F_(2,24)_ = 0.13, 0.87	F_(6,35)_ = 0.02, 0.99
			dNCM	3.08	0.15			
			vCMM	3.35	0.69			
			vNCM	1.13	0.08			
		hSyn1 (GFP)	dCMM	6.38	1.76			
			dNCM	2.80	0.34			
			vCMM	3.19	0.52			
			vNCM	1.17	0.07			
