# Supplementary material for: A reliable and flexible gene manipulation strategy in posthatch zebra finch brain
Source: Sci Rep. 2017 Feb 24;7:43244. doi: 10.1038/srep43244 (PMC5324116; doi:10.1038/srep43244)
Supplement: Supplementary Tables [file srep43244-s1.pdf]

SUPPLEMENTARY INFORMATION

A reliable and flexible gene manipulation strategy in posthatch zebra finch brain

Somayeh Ahmadiantehrani and Sarah E. London

Table S1: Cell counts in AF subregions – Effect of Subregion

Measurement	Age	Subregion	Mean	SEM	main effect	P-value
FP+ Cells	P30	dCMM	11.68	4.88	$F_{(3,12)}$=0.52	0.68
		vCMM	12.54	4.95		
		dNCM	8.99	3.01		
		vNCM	6.22	2.30		
	P40	dCMM	22.80	3.79	$F_{(3,8)}$=1.08	0.41
		vCMM	26.40	3.21		
		dNCM	20.13	4.28		
		vNCM	17.13	3.81		
	P50	dCMM	20.80	5.69	$F_{(3,8)}$= 0.49	0.7
		vCMM	24.40	5.69		
		dNCM	17.00	5.69		
		vNCM	15.73	5.26		
NeuN+ Cells	P30	dCMM	253.99	7.76	$F_{(3,12)}$=0.27	0.85
		vCMM	250.93	3.96		
		dNCM	253.29	6.65		
		vNCM	246.79	6.02		
	P40	dCMM	238.93	2.79	$F_{(3,8)}$=1.22	0.36
		vCMM	249.47	4.11		
		dNCM	257.27	3.71		
		vNCM	252.87	12.75		
	P50	dCMM	247.60	4.91	$F_{(3,8)}$= 0.79	0.53
		vCMM	246.87	5.04		
		dNCM	257.40	2.72		
		vNCM	259.47	12.64		
DAPI Nuclei	P30	dCMM	573.52	26.49	$F_{(3,12)}$=0.01	0.99
		vCMM	574.70	18.31		
		dNCM	570.77	21.03		
		vNCM	577.59	26.32		
	P40	dCMM	601.47	8.63	$F_{(3,8)}$=0.11	0.95
		vCMM	603.20	7.76		
		dNCM	600.73	9.16		
		vNCM	596.67	7.65		
	P50	dCMM	605.73	15.11	$F_{(3,8)}$= 0.82	0.52
		vCMM	612.00	3.46		
		dNCM	589.87	14.06		
		vNCM	596.13	5.96		

Table S2: Cell counts in AF subregions – Effect of Age

Measurement	Age	Subregion	Mean	SEM	main effect	P-value
FP+ Cells	P30	dCMM	11.68	4.88	$F_{(3,12)}$=0.52	0.68
		vCMM	12.54	4.95		
		dNCM	8.99	3.01		
		vNCM	6.22	2.30		
	P40	dCMM	22.80	3.79	$F_{(3,8)}$=1.08	0.41
		vCMM	26.40	3.21		
		dNCM	20.13	4.28		
		vNCM	17.13	3.81		
	P50	dCMM	20.80	5.69	$F_{(3,8)}$= 0.49	0.7
		vCMM	24.40	5.69		
		dNCM	17.00	5.69		
		vNCM	15.73	5.26		
NeuN+ Cells	P30	dCMM	253.99	7.76	$F_{(3,12)}$=0.27	0.85
		vCMM	250.93	3.96		
		dNCM	253.29	6.65		
		vNCM	246.79	6.02		
	P40	dCMM	238.93	2.79	$F_{(3,8)}$=1.22	0.36
		vCMM	249.47	4.11		
		dNCM	257.27	3.71		
		vNCM	252.87	12.75		
	P50	dCMM	247.60	4.91	$F_{(3,8)}$= 0.79	0.53
		vCMM	246.87	5.04		
		dNCM	257.40	2.72		
		vNCM	259.47	12.64		
DAPI Nuclei	P30	dCMM	573.52	26.49	$F_{(3,12)}$=0.01	0.99
		vCMM	574.70	18.31		
		dNCM	570.77	21.03		
		vNCM	577.59	26.32		
	P40	dCMM	601.47	8.63	$F_{(3,8)}$=0.11	0.95
		vCMM	603.20	7.76		
		dNCM	600.73	9.16		
		vNCM	596.67	7.65		
	P50	dCMM	605.73	15.11	$F_{(3,8)}$= 0.82	0.52
		vCMM	612.00	3.46		
		dNCM	589.87	14.06		
		vNCM	596.13	5.96		
